# Supplementary material for: Strategies to build trust in the conduct of clinical trials: Stakeholders’ views in a qualitative study in Ghana
Source: PLOS Glob Public Health. 2025 Apr 8;5(4):e0003201. doi: 10.1371/journal.pgph.0003201 (PMC11978029; doi:10.1371/journal.pgph.0003201)
Supplement: S1 Data — (DOCX) [file pgph.0003201.s001.docx]

**Name:** Strategies to improve trust and participation in clinical trials

<Internals\\IDIs\\A-Participated 3 or more times\\B-IDI-31yr old female-participated 3 or more times-02> - § 1 reference coded [2.31% Coverage]

Reference 1 - 2.31% Coverage

Q: What do you think cold be done to improve trust and participation in such studies?

R: For me, if you go into the communities and sit with community leaders and the people and explain to them about the work they will understand.

<Internals\\IDIs\\A-Participated 3 or more times\\C-IDI-60yr old female-participated 3 or more times-03> - § 1 reference coded [5.17% Coverage]

Reference 1 - 5.17% Coverage

Q: You have explained the factors that make people not interested in taking part in our trial studies. In this community, what do you think cold be done to improve trust and participation in such studies?

R: Left to me along, I think you have to intensify your education and let people understand your work especially those who do not have interest in your work. Am sure you have their names in your books and if you can identify them and go to them and explain for them to understand your work just like I have understood it, I think that will help.

<Internals\\IDIs\\A-Participated 3 or more times\\D-IDI-30yr old femlae-participated 3 or more times-04> - § 1 reference coded [5.06% Coverage]

Reference 1 - 5.06% Coverage

Q: What do you think we can do to improve trust and participation in trial studies in this community?

R: For that one, I think if we are able to sensitize or educate everybody and let them understand the essence of the work and that it has no effects on their health that will improve participation

Q: What else?

R: And the benefits of the drugs, they should explain all those things to people and I think if that is done people will have trust on the work.

<Internals\\IDIs\\A-Participated 3 or more times\\E-IDI-52yr old female-participated 3 or more times-05> - § 2 references coded [14.83% Coverage]

Reference 1 - 6.77% Coverage

Q: Don’t you think community education will help address these perceptions?

R: Well, if that is done some will understand but other will not still understand. I have made them to know that for instance, if you fall sick and go to the hospital and they take your blood, do they go and sell it? And it is the same things the research people do. You will say all these to them and yet they don’t want to understand. So maybe you people can go and talk to them and see.

Reference 2 - 8.06% Coverage

Q: Would you say that there is lack of community opinion leaders’ involvement in our work and that is why some people have these perceptions at the community level?

R: Well, they have been talking to us at the clinics because there is no clinic that you will visit and you will not see research workers there. So there have been educating them but that is also their perception and what can you do. However, you need to continue to educate them and those who can change will change and those who will not understand, they should also have their opinion.

<Internals\\IDIs\\B-Refused and dropped outs\\F-IDI-28yr old female-Dropped out-01> - § 4 references coded [14.69% Coverage]

Reference 1 - 5.12% Coverage

R: For me if you are doing something with people just be sincere so that it does not look like you are just using something to convince me to join. If you can do one thing just say that this is what we can do and when you do it, it will make people to be interested in what you are doing. If you have so many things stated and you cannot even do one of the things that is not good even though that might not be the reason why you are joining but it will look at if there is no seriousness in what they are doing because then even if my child is seriously sick you will still waste my time (use this quote).

Reference 2 - 3.50% Coverage

they should try and fulfill some of the things they normally say they will do to motivate people to continue to be part of their work because we are all helping each other and if we (study participants) are not there they (researchers) will not also be there and the vice visa. At least the little time you take from us (refers to trials participants) we should gain something small from it, yeah (use this quote).

Reference 3 - 2.33% Coverage

So the point I am making is that those that you recruited if you motivate them it will make others to also join and if you are not doing that it will make many people to know that you don’t fulfill your promise. Those who will drop out can even tell other people not to join.

Reference 4 - 3.74% Coverage

R: For me it is the honesty I will still talk about and what I will say is that they should try and fulfill some of the things they normally say they will do to motivate people to continue to be part of their work because we are all helping each other and if we are not there they will not also be there and the vice visa. At least the little time you take from us (refers to trials participants) we should gain something small from it, yeah.

<Internals\\IDIs\\B-Refused and dropped outs\\G-IDI-23yr old female-Refused to take part-02> - § 1 reference coded [2.91% Coverage]

Reference 1 - 2.91% Coverage

R: They should try and fulfill their promises and they should also talk to their workers to know how to talk, yea.

<Internals\\IDIs\\B-Refused and dropped outs\\H-IDI-32yr old Female-Refused to take part-03> - § 2 references coded [8.99% Coverage]

Reference 1 - 3.44% Coverage

R: I would suggest that before any intervention that would take place at the community level, they should try and educate people very well on it before they introduce it. The people who are even going to do the education should be well trained to know how to do it.

Reference 2 - 5.55% Coverage

So your people have to be patience in their dealings with community members and also they should know how to handle educated people and also how to handle those who are not educated. For example, it is more difficult to deal with those who are educated and so they always have to take their time and explain the things for them to understand and not trying to argue with them especially when they ask questions for clarification.

<Internals\\IDIs\\C-Opinion leaders-Nav\\L-IDI-53yr old male Assembly Member-Nav-01> - § 4 references coded [6.06% Coverage]

Reference 1 - 1.49% Coverage

So I want to say that in other to overcome such a challenge, you need to really maintain and let people know where and how you started so that if fault people are also entry into the community, people will be able to make that distinction and know that they are not the people they have been working with.

Reference 2 - 0.97% Coverage

So in order to make people trust the research that we go through then the researchers must be well resourced and equipped so that they can really carry out very tangible research, yes.

Reference 3 - 0.80% Coverage

So when people are really compensated in a way, they are highly influenced and the trust is built. So we can’t run aware from it because those days are past, laughter.

<Internals\\IDIs\\C-Opinion leaders-Nav\\M-IDI-47yr old youth leader-Nav-02> - § 1 reference coded [11.26% Coverage]

Reference 1 - 11.26% Coverage

Q: What do you proposed could be done to improve trust and participation in the conduct of clinical trials studies in Ghana?

R: I think you need to continuously educating the people about the importance of your research activities. You see it? And what I will propose is that of you can establish a radio station at your research centre mainly to propagate your research activities in the local languages; it would help people to have better understanding of your research work before you can make a follow-up to continue with the community level education. This can also be an opportunity for you the research people to educate the community members on health issues and that for me would go a long way to improve health status of people in the area.

<Internals\\IDIs\\C-Opinion leaders-Nav\\N-IDI-Paramount Chief-Nav-03> - § 2 references coded [6.21% Coverage]

Reference 1 - 2.32% Coverage

R: That is why I am saying the education is always the problem. if the education is not enough just as some people have earlier on said, you come, you introduce the study to them, that this is the study you are coming to make and that you will be sending field workers to come round and this is what they are coming to do. So, when they come, you will be aware of them, they will becoming this day and they will be ending on this day and you think that is enough and you go back. At least once in a while before the study period elapses you have to come back and remind the community of what you said and that the fieldworkers are on working. So anyone who doesn’t understand them should ask where he/she is finding it difficult to work with them and hear from the people.

Reference 2 - 3.88% Coverage

Q: People have complains that the fieldworkers sometimes they ask questions and they are not able to address the questions so like you are saying it is important for the main people who are leading the study to from time to time go to the community, organize durbars and let them ask. (Interruptions by the respondent).

R: Oh yes! That is what I am saying to know the difficulties whiles your fieldworkers are in you are calling to find out if they are finding some difficulties with your fieldworkers. Or the problems they are facing with your fieldworkers, they want to know whether it is from the fieldworkers or from you the community members so that you can address them so the study can go on smoothly. But if that is not done you see those who have that perception will say oh it is true if not they would have come to explain to us because after introducing the study to them, they are aware some fieldworkers will be coming from this day to this day. But while the study is going on difficulties must be identified and if you don’t go round to find out whether there are difficulties and what the difficulties are and how we are solving the difficulties or meeting them so that the study can end up smoothly, by all means the study must end up crookedly. (Broke into laughter).

<Internals\\IDIs\\D-Opinion leaders-Hohoe\\O-IDI-60yr Old male Assembly Member-Hohoe-01> - § 3 references coded [10.25% Coverage]

Reference 1 - 3.18% Coverage

Q: okay so what do you think could have been done differently to facilitate easy access?

R: to facilitate easy access the thing that is why we need to first of all have education, anytime you want to do something there is always an education. Educating them why there they have to allay their fears, so that finally they will give themselves. When they give themselves up and on many occasions people want to say well at least if you are going to do this thing, what are you giving me, what will I gain out from it? Well, I don’t know whether tomorrow I will die because of this so they think of this say, so we need to motivate!

Reference 2 - 2.14% Coverage

Q: the time factor too?

R: the time factor because the awareness must be created enough, and there should be somebody out there to actually give them a comfort zone that oh I am there, I know what it is, it’s nothing, there is a focal person, the link you between the people like an opinion leader and you should there should be a good rapport between you and the community so it also depends on who the opinion leader is.

Reference 3 - 4.94% Coverage

R: which is no. you see, they had that culture that anything that government proposes or anything that is new, they expect the honorable member to be aware of it. So when they are less privilege to have knowledge about it they normally tend to ask from the honorable person “yeah we hear the government doing this, we hear people coming to say this, how what do you know about it and we also have our culture that anytime anybody wants to come to the community , he should pass through the focal person so when the person goes for instance if you come to see me and I allow you to go round doing this, people who don’t know you or probably might not be available the time we made the announcement they will begin to find out that there is somebody going around. Do you know anything about it? So definitely the focal person or the link person should be aware of the very programme and know the details of it so that when they come. So as they come we give them that explanation.

<Internals\\IDIs\\D-Opinion leaders-Hohoe\\Q-IDI-59yr old male Assembly Member-Hohoe-03> - § 1 reference coded [1.96% Coverage]

Reference 1 - 1.96% Coverage

R: yes when it comes to that level, it is very important that the stakeholders like the chiefs and then assembly members, the unit committee if you involve them then you are through, yes because they believe in the chiefs, assembly and the community and then other stakeholders in town hall meetings or whatever it is, it works perfectly but without them there will be a lot of questions yes they will not accept you in the community.

<Internals\\IDIs\\D-Opinion leaders-Hohoe\\R-IDI-60yr old male opinion leader-Hohoe-04> - § 2 references coded [9.93% Coverage]

Reference 1 - 3.91% Coverage

R: ok to talk about trust, once I see the credentials of the researchers then I can have hope in what they are about to do and be able to say how honest they will be and also when education is done very well then I think it affects the trust of conducting clinical trials.

Reference 2 - 6.02% Coverage

1: What do you think are the necessary steps or procedures that could be followed to improve the level of trust and participation in clinical trial studies in Ghana or in this community?

R: Firstly if they want to come they should come and educate us, they should come and see the chiefs and does community engagement so we all understand what the whole thing is about, only then will we take part in clinical trials.

<Internals\\IDIs\\D-Opinion leaders-Hohoe\\S-IDI-64yr old Sub-Chief-Hohoe-05> - § 1 reference coded [4.46% Coverage]

Reference 1 - 4.46% Coverage

R: There should be a general education in the community, they have to tell the chief and elders then the whole community will also be educated on the importance of the trial and I think that would improve participation and trust in clinical trials.

<Internals\\IDIs\\D-Opinion leaders-Hohoe\\T-IDI-male-Forma Director of health services-Volta Region-06> - § 1 reference coded [5.75% Coverage]

Reference 1 - 5.75% Coverage

R: I think that we should carry out public awareness; we should not wait to have a trial before we think of educating the people about it. People must know that clinical trials are very, very important and we may be doing clinical trial in the country as and when necessary. Sometimes, we take thing for granted that our people are not educated and they may not understand these issues but when you educate the people very well they will understand. So generally, I think that education in the country is very low even in all areas and so I think we should take it up and create awareness of the conduct of trial studies, the importance of clinical trials among the general public. Once we are able to create awareness I think things will be easier than it is now. The researchers must also be educated to do the right thing like how to administer the consent form you know it should be done in a language that the people understand. They should explain all the study procedures very well to participants before they can decide whether or not to take part in the study.

<Internals\\IDIs\\E-community members-Hohoe\\V-IDI-57yr old female community member-Hohoe-01> - § 3 references coded [15.13% Coverage]

Reference 1 - 6.02% Coverage

1: What do you think are the necessary steps or procedures that could be followed to improve the level of trust and participation in clinical trial studies in Ghana or in this community?

R: I think once these issues come up those in higher authorities should first come to inform us, they should involve the chiefs, they should do something like community engagement and then educate we the community members as well then we can have confidence in the trial.

Reference 2 - 7.11% Coverage

I: What are the best mechanisms or strategies to facilitate the conduct of clinical trial studies in this community/ Ghana? Probe for many examples

R: one thing we can do we should build trust, we should explain to the participants what the risks and benefits are in the trial studies and tell them the details of what the trial is about. In terms of education, proper education should be given to people so people really understand what they are been involved in so they can decide for themselves whether to take part in such studies or not.

Reference 3 - 2.00% Coverage

R: education is very key, so when these issues happen the authorities should be quick to respond and send information to us so we are also aware of them.

<Internals\\IDIs\\E-community members-Hohoe\\W-IDI-29yr old male community member-Hohoe-02> - § 2 references coded [11.65% Coverage]

Reference 1 - 5.56% Coverage

R: I think once education is done well and am fully convinced then I can take part. Also the risk and benefit should be well explained so we know exactly what to expect when we take part in this type of trials. Also compensation should be given because you don’t expect someone to just take part in a study without promising the person anything then some will not take part in the study. For example is when you go to donate blood you are given some provisions to regain your blood back, it’s the same for trials as well.

Reference 2 - 6.09% Coverage

1: What do you think are the necessary steps or procedures that could be followed to improve the level of trust and participation in clinical trial studies in Ghana or in this community?

R: As I said earlier the opinion leaders should be involved in the decision making process, secondly the opinion leaders should organize a durbar for the whole community and educate them on the trial giving them assurance of what to expect from the trial will boost the confidence of the people. That I think will go a long way to build trust and making the community also partake.

<Internals\\IDIs\\E-community members-Hohoe\\X-IDI-44yr old female community member-Hohoe-03> - § 2 references coded [2.87% Coverage]

Reference 1 - 2.26% Coverage

R: I think once education is done well and the people understand what is going on then that could motivate them to partake.

Reference 2 - 0.61% Coverage

R: The chiefs should be involved.

<Internals\\IDIs\\E-community members-Hohoe\\Y-IDI-49yr old male community member-Hohoe-04> - § 1 reference coded [3.24% Coverage]

Reference 1 - 3.24% Coverage

R: I think the health care professionals should first come to the community and talk to the chiefs of the community and then the whole community would be invited to be educated, then that will be the right strategy to influence the community.

<Internals\\IDIs\\F-MPs\\Z-IDI-47yr old MP-01> - § 3 references coded [40.36% Coverage]

Reference 1 - 16.13% Coverage

Q: What is your perception on the conduct of clinical trial?

R: There are various stages that clinical trials go through. They first stage is where the drug is being tried on animals to see how it works before it is tried on human beings or people because when you first try the drug on human being and once you loss a life, you cannot bring it back and so in conducting clinical trial studies, you make sure that people don’t loss their life or get other disease as a result of their participation in the trial. Therefore, there should be a community communication strategy to educate people that it is safe for them to take part, the drug you are going to try is safe for people to use because once people are not sure of its safety, it will be difficult for them to take a decision to get involved. So communication is key when it comes to the conduct of clinical trial studies because people need to understand what you are doing and they must be convince that it will not be harmful to them when they take part and when people have that belief, they will accept it.

Reference 2 - 8.20% Coverage

R: That is why I earlier on indicated that researchers must show that it is safe for people to get involve in whatever trial they want to undertake by showing pictures and assure people that it has been done in other places and there are the negative or positive effects. The issue is that it is a very high risk for you to take part in trial studies especially, phase one clinical trials. So for me, I will not even take a decision to take part in the initial stages of the trial (refers to phase one trials) because of the high risks involve.

Reference 3 - 16.03% Coverage

R: Assure the people that what you are going to do is beneficial to them and once the people are assured that it is safe and not harmful to them, they will participate. The other issue has to do with education. When the people are made to know what exactly you are doing, the benefits and the risks involve through appropriate community sensitization and stakeholder engagement, I believe it will boost the level of trust. Tell them the truth about the negative and positive effects of it and allow them to take the decision whether to take part or not. Instances where you only project only the positive aspect of it without letting them also know the risks or the negative aspects, it is dangerous because one person along can spread information if the person gets involve and experience anything differently and once that person goes to tell other people before you know the information is everywhere and you can imagine what will happen to whatever you are doing. So telling the truth about whatever you are doing will help build level of trust in my view.

<Internals\\IDIs\\F-MPs\\ZZ-IDI-54yr old MP-02> - § 1 reference coded [2.86% Coverage]

Reference 1 - 2.86% Coverage

R: You will understand that what you are being tested or used for would one day benefit the larger society and so that is an aspect that if you explain very well you would get people to be involved. You also need to convince people that it will not affect them in anyway and if there are even side effects they are going to be temporal, if we give you this vaccine you may get headache, your temperature may go high and all these things will come down and when you are able to convince people that way I believe people will be interested to take part.

<Internals\\IDIs\\F-MPs\\ZZZZ-IDI-49yr old MP-04> - § 2 references coded [12.03% Coverage]

Reference 1 - 3.68% Coverage

**Use text** (solution to improve conduct of trials)

Therefore, on my part if there is any recommendation to be done *eeem* as we move alone there should be that vigorous public education and not only waiting till there is a clinical trial to be done but it should be part of our public education process to educate people about the whole process of clinical trials so that people are aware that when you see that they are conducting clinical trials it is not only to harm our people but the benefits are there. We can give examples that they were so many diseases in the past but today you don’t see them and it is because other countries took part in trial studies that we used their vaccines without fear and so if we don’t also subject ourselves to it then *aah* we would not be contributing to the scientific advancement.

Reference 2 - 8.35% Coverage

Q: What can we do in Ghana to improve trust and the conduct of clinical trials?

R: We can do a number of things like the education that I mentioned earlier, we do evidence based education and not just *eem*, *eem* going to radio or TV and talking about it no. Science has advanced and we know that’s how the 6 killer diseases came to be because they (6 killer diseases) were really killing children. So we have evidence that no the benefits of vaccines are great and very helpful to human beings. So if we go and start from there and get a number of vaccines that we know have been used for years and let people know how they were developed…use that to educate the people because many of the vaccines or drugs we use in Ghana were bot developed in Ghana even in Africa, so we just sit and then they develop and bring them. So if they know that some human beings also availed themselves before we get those drugs or vaccines then they know that *oh* we are not the first to be used you know in clinical trials and we raise their level of trust by assuring them that your fears of what you (researchers) do are not what would happen. Is all about the fear of the unknown and using your scientific knowledge and skills, you can at least relief or bring down these anxieties just by explaining things to the people. Let’s say in a period of a year, even if it is quarterly or some period of the year you go to the media stations, churches, mosque and we begin to explain to our people what clinical trials are all about, the process and all that of course at a point we will know that oh it’s (trials) something that is there and we also need to get involved in it. The way it is now, we are used to getting the final product and we do not really know the processes it goes through and so the public education about clinical trials is very important.

<Internals\\IDIs\\F-MPs\\ZZZZZ-IDI-49 yr old MP-05> - § 8 references coded [26.21% Coverage]

Reference 1 - 1.18% Coverage

So should it be with this trial, you don’t take the advantage of the ignorance of the people just come around, hide what you really want to do and pay some peanuts and then ignorantly take their data and then when there is something wrong there is no fallback position.

Reference 2 - 1.86% Coverage

my view is that we need to have a protocol, I mean have a certificate that you will get people who understand the language of the person that are either taking the sample or you are giving the medication or whatever to explain what is going to happen to the person in the way that the person understands and be frank with the person the likely consequences so that the person is in the known and then compensate adequately.

Reference 3 - 5.55% Coverage

R: It is very low because of the credibility gap, in the past people have abused people they have come into community, not entering the community properly, not educating the community people properly, not telling them exactly what the consequences of the things are and sometimes it is distasteful so it went quite, sometimes back fired and it has created problems. So today when you come and you are talking they want to be sure is it all the information I need to know or you are hiding something so the suspicion is very high and I think the only way to eradicate this suspicion is to be consistent and truthful. And we as a country and for that matter ministry of health must have a protocol that is known to the people so that I know that once it is going to be a trial in my constituency I expect A, B, C, D, E, F is going to happen. Once I know that it will happen in Asewase, it will happen in Bolga, it will happen in Zebila, the same thing is happening in Ho, the same thing is happening in Kpando, or Sefwi or somewhere. Then this suspicion will be off but if you enter the community differently and behave differently and give half information, in some cases you give full information, the protocol is not well known definitely the suspicion will remain.

Reference 4 - 3.99% Coverage

R: No, sometimes your choice of community I remember very well that when they wanted to do the Ebola one they chose the Volta region and that time the speaker of parliament was a voltarian and his question was that why did you choose the Volta region? Are you getting it, why Volta region? Of all the regions, why Volta region? So for me, what I will say is that if you want to do this trial, let’s say you need fifty people, why not locate it in three places instead of just one, are you getting it? Because we are in a country for example in our society, very tribalistic, religious, all the other things, people are very conscious about what is happening in their community so it is a bit of this thing but when you do it and there is a mixture maybe urban, rural, then it is like oh not because we are rural people they want to cheat us. I think that these are some of the things that need to be factored in.

Reference 5 - 2.61% Coverage

R: You see when ebola comes and even by just starting an ebola person this is the consequences you need to educate them properly. Educating the people properly about why you want to do it, what are the benefits the consequences would take more time and energy than if there was not an outbreak because everybody saw the consequences of the outbreak in Sierra Leone and Liberia. And obviously you just mention the name and it is now to scare people to their spine so I think that, that was partly the reason because of the escalation of the news about the terrible effect of the ebola and all that.

Reference 6 - 3.83% Coverage

R: That is why I said proper education, entering properly, getting the community leaders involved, getting the village champions to appreciate what it is all about, telling them what the consequences are likely to be and when it does happen, what will be the fallback position, how should they be able to rescue them, that should be very clear. Even me sitting here I support trials but if you come and tell me you want to do trial on ebola, I would ask thousand and one questions. I want to be convinced beyond reasonable doubt that you are not coming to introduce the ebola virus in me so definitely you needed to do the entering of the community, they didn’t need to rush. Yes, I heard that they have been doing for three months but because of the escalation of the news on ebola, they needed more time and more strategy in handling it than I imagined they wanted to do it.

Reference 8 - 5.13% Coverage

R: We need to do mass education and the ministry of health in particular needs to properly advertise and educate before on its protocol. Oh if people want to come and do trials in Ghana these are the things that you expect them to do so that the community members know. They use it as a checklist so when you enter, they will be expecting you to do A, B, C-F so if ministry of education does that and makes it part of its educational programmes, these drama and other things popularize it so that people know. When MTN wants to introduce a **momo** with this “momo” look at how they advertise, now a lot of people are keeping wallets. You need to educate people to understand the benefit of what you want to do you cannot just assume because
