# Supplementary material for: Strategies to build trust in the conduct of clinical trials: Stakeholders’ views in a qualitative study in Ghana
Source: PLOS Glob Public Health. 2025 Apr 8;5(4):e0003201. doi: 10.1371/journal.pgph.0003201 (PMC11978029; doi:10.1371/journal.pgph.0003201)
Supplement: S2 Data — (DOCX) [file pgph.0003201.s002.docx]

**Name:** Strategies to improve trust and participation in clinical trials

<Internals\\KIIs\\A-Clinical trials researchers\\B-KII-48yr old trial coordinator-02> - § 2 references coded [7.71% Coverage]

Reference 1 - 3.07% Coverage

Q: If the level of trust is low at the community level don’t you think that we have a challenge because they are supposed to get involve in these trial studies and if their level of trust is low then it affects the conduct of trial studies. Don’t you think so?

R: I think this work will then inform the Scientists that don’t just sit in the big rooms and speak the big English. You need to break the English down for the community people to understand. You know most of these community people how many of them can read and understand? How many of them can read posters and understand what is there and how many people listen to news and you see even in the news, it is big, big English and they will not understand. I think, at the community level, we need to identify the lowest level of communication and apply it there for them to understand otherwise this mistrust will continue to be there.

<Internals\\KIIs\\A-Clinical trials researchers\\C-KII-58yr old clinical trial investigator and Director-03> - § 1 reference coded [1.77% Coverage]

Reference 1 - 1.77% Coverage

Give the people the full story on the benefits and the side effects of what you are going to do, let them know the risks and benefits ratio, let them know what its entail to be in the study, if you are going to take blood seven times, let them know and don’t just say you will take blood so that they can take informed decision. Also let them know that even if they refuse to take part there will not be any punishment to them in anyway and when you do all that I think the trust will be there.

<Internals\\KIIs\\A-Clinical trials researchers\\E-KII-48 yr old clinical trial Monitor-05> - § 3 references coded [8.45% Coverage]

Reference 1 - 3.39% Coverage

Q: Do you think education in that regard can influence their trust positively for them to get involve in trial studies that take blood samples.

R: Yes, consenting process has to be done very well and not just telling people what the study is all about but you know going outside to let them know why the trial is being conducted, the importance of it and then they contributing their time in order to allow the trial to be done so that we will be able to find drugs or vaccines to help humanity. So education and on the need to conduct these trial studies and then consenting process should be done very well in order to build trust of community members.

Reference 2 - 1.01% Coverage

Q: What can we do to influence trust positively in Ghana when it can to the conduct of trial studies?

R: I think education is key for us to do, give them the information and allow them to decide.

Reference 3 - 4.05% Coverage

Q: If you are saying they should do the education, where do you think should be their starting point? At the community level of they should engage key stakeholders?

R: I think the education can take different forms, they could go and speak on radio in a particular community that we have approved this trial, it is like this, it is like that, we have looked at it and we have approved it and we think that it will help us and so they will be coming to the community and if they talk to you and you can take part fine. You know the radio riches far at the community level and they could use that but the issue is who will provide the funding for that activity? They don’t charge much in their review processes and the funding will be a problem for then to undertake such an activity.

<Internals\\KIIs\\A-Clinical trials researchers\\G-KII-55yr old clinical trial investigator-07> - § 3 references coded [8.22% Coverage]

Reference 1 - 4.53% Coverage

There is the need for a general education or discussion on how clinical trials are conducted and why there is the need for clinical trials to be conducted. You mention the ebola trial that was going to be conducted in a place where there has been some level of trial but the issue was at that time ebola was more of a killer disease and also people did not understand the trial that was to be conducted and that was the reason why it had to go all the way to the level of Parliament. Trust is important in clinical trials but the key thing is that once you begin to talk about trust eeem, people should also understand what is going into the trial, why it is being done? How it is being done? Who is involved? And what will be the outcome of the trial.

Reference 2 - 1.05% Coverage

However, I would urge investigators to stick to the processes that have been outlined in the protocol and the consent form. Once that is done, all these mistrust would be minimal.

Reference 3 - 2.64% Coverage

So the lessons I have learned from this issue is that we don’t take anything for granted. A mathematician who is a member of Ghana Academy is a mathematician, a Historian is a Historian *eem* an epidemiologist is an epidemiologist and so we don’t have to take things for granted that *eem* an epidemiologist who has done epidemiology about diseases should therefore know about the conduct of clinical trials. So, that engagement needed to have been done.

<Internals\\KIIs\\B-Ethics members\\G-KII-54yr old male ethics Ccommitteemember-Nav-01> - § 2 references coded [16.71% Coverage]

Reference 1 - 9.67% Coverage

Q: What would you recommend should be done to influence trust positively and for people to get involve in trial studies in Ghana? Now that you have highlighted the challenges, what do you suggest to be done to improve the conduct of clinical trial in Ghana?

R: Yes, and so that is a very good one, so if you use the ebola trial as an example, if clinical trials are going to be conducted either Ghana or anywhere the first step is to get the politicians involved. Before you even say *eeeh*, *eeeh* you go into that venture you would have informed the Government … I think they do that you would have informed the Government, *eeem*, the executive, you would have informed Parliament, you would have educated those people and let them know the level of trust you want to have on them. If you don’t educate them and they start talking, you know, you will explain something to me and the way I will go and explain it to the second person may not be the way you have explained it to me. What I’m trying to say is that you have to make sure those people (refers to politicians) are properly educated as far as that trial is concerned so that they will not misinformed anybody. You have to let them know the time you would want them to go and communicate to their people and you make sure that until they are properly educated and they understand what is involved, you will not let them go and communicate to their people. If you do that I don’t see the reason why people should have problems with it.

Reference 2 - 7.03% Coverage

It doesn’t means that if you educate them, everybody is going to come on board, even in the US where they conduct such trials, there is a procedure, you have to fill a form and they say they will give you this amount of money should in case you die and all those things and then you consent for it. So, it is not force, in clinical trials, you don’t impose pressure on anybody. They educate you and if you are interested you join. So, if you scare them, you will not get anybody coming to take part. So, it is important to do the education and it should start from the top, the government, to the parliamentarian and them to some of us such as Universities, colleges and then to community members. If you educate all of them properly, I don’t see why you would not get people to take part in the trial.

So stakeholder engagement is important and the way you go about it is very, very critical. As I said get people to understand what you want to do before they intend telling people about it. If they don’t understand, they would spread wrong information about it and that may bring about mistrust.

<Internals\\KIIs\\B-Ethics members\\H-KII-47yr old male ethics committee member-Nav-02> - § 1 reference coded [3.43% Coverage]

Reference 1 - 3.43% Coverage

Q: ok so as a member of the ethics committee what do you think should have been done as far as that particular trial is concerned and then other trials you know, what do you think should be done to improve the conduct of clinical trial in Ghana taking into consideration the ebola trial?

R: maybe once it’s a clinical trial, they should involve all the political parties in Ghana especially the major ones no matter what it is almost like 50|50 so if we engage them earlier in advance and then they are convinced to engage those who will publicize whatever you are doing we have been, we have like the media very well, it will help and there are other influential leadership in the country who are stakeholders, other stakeholders must be met in various capacities so when such major groupings are met and they un…they understand the whole process when there is an issue they can even help to defend.

<Internals\\KIIs\\B-Ethics members\\I-KII-42yr old female ethics committee member-GHS-03> - § 2 references coded [12.05% Coverage]

Reference 1 - 9.34% Coverage

R: It is all about the education, you know if you are not able to explain your information sheet well to the people (not able to administer informed consent well), if you are not able to do your community entry very well, if you are not able to explain the issues in the language that the person understand to enable them take informed decision then that is where the problem is. So even with the normal protocol, if you are not able to explain the issues for the person to know whether he/she should take part or not then that is where the problem. So the information sheet is very important and it depend on how simple it is and how people are trained to explain the study procedures to the participants to really understand and that is what is going to build the trust. So for instance if you give the information sheet to the person to take home and discuss with another person to probably build on his/her interest then depending on the person’s view on that thing (refers to the study) and what the person has heard before, if there is so many misinformation about it, that is where they might not be trust or something has happened in the community before and they did not understand it well and that is still in their minds and when you go there with a new study, based on previous experience then that is where the trust might not be there. For instance the Ebola thing (ebola vaccine trial) there were so many hullabaloo (so many noise about it) and people who were even educated did not understand what the study was all about, people did not understand the whole process and when it happens like that and the information gets out there to the community, everyone will interpret it the way he/she wants and that brings about the mistrust.

Reference 2 - 2.72% Coverage

But I think if you do your community entry well, if you do your information sheet such that the information is so simplify everyone will understand it easily and I think that will build trust of people to get involved in whatever you are doing. The other thing too is about the study procedure, what do you want the study participants to know and that is one other point and so if that is not clear, everyone will be interpreting is the way they understand it and that will bring about confusion and mistrust.

<Internals\\KIIs\\B-Ethics members\\J-KII-59yr old male ethics committee member-GHS-04> - § 2 references coded [2.49% Coverage]

Reference 1 - 1.53% Coverage

We encourage researchers that their community entry should be very important, they have to be very visible to the community, fins opinion leaders and educate them about the whole process and what they want to do in the community and even make it continues before the trial takes place. Without that, it is very difficult to earn the trust of the community.

Reference 2 - 0.96% Coverage

Suggestion

I think one of the things we should have done was to really, really, really have a section with the media, charged them the responsibility and then pay them to go and educate the people that though we said earlier but now these are the facts of the trial.

<Internals\\KIIs\\B-Ethics members\\K-KII-51yr old female ethics committee member-Nav-05> - § 5 references coded [13.61% Coverage]

Reference 1 - 3.79% Coverage

R: We do a lot of community engagement here (refers to Navrongo) before we start the trial and of people are not interested and they don’t want to take part, you can’t force them. However, you have to give them as much information as you can because usually before the trial starts and before the informed consent, you have to do community engagement where you involve the chiefs and opinion leaders at the community level. Then before the participants will even be made to consent, you go to their homes, invite them and explain… the gatekeepers, the household heads and all those people and they ask all the questions that they have. So them if you are administering the informed consent, it is not something that is new to them. Apart from that we have some key community members who usually help us to address issues at the community level.

Reference 2 - 1.47% Coverage

For us, we keep our promises, yeah. Some people might have taken part in a previous study that promised them something and so, usually, when they tell you, you try to find out which study was that and then you go back and tell the investigators about it and sometimes, they also go back and explain to them.

Reference 3 - 2.17% Coverage

Q: So based on what you have just explained, do you think we still have a problem in Ghana regarding trust and the conduct of trial studies?

R: Yeah and I think there is still the need for more education and the education should target everybody including key stakeholders at the national level and that is why I said the level of trust and participation in trial studies is high in Navrongo because the people are well informed and they know that there are rules that are followed.

Reference 4 - 1.55% Coverage

R: Apart from the education, I don’t really know what other factors can affect the conduct of trial studies. Whoever is conducting trial study has to conducting it strictly according to GCP and should not cut corners. You just do the right thing all the time and also educate people all the time about the trial and their rights as participants.

Reference 5 - 4.62% Coverage

Q: Well, what would you suggest to be done to facilitate the conduct of clinical trials in Ghana?

R: So, what I will say is that clinical trial researchers have to stick to the rules that govern the conduct of trial studies and they should not cut corners. The issue is that it has to do with integrity so you have to have the highest integrity and you consider the safety of the subjects as the most important thing and not maybe the results that you are going to get. The safety of the participants should be what you look out for and not your personal ambitions like your paper that you will publish, your money your career and all of that because in the first place you are doing the trial in the interest of humanity and that is what you should think about. Also, there should be more education even when there is no clinical trial, there should be constant education on clinical trials because anybody can just go on social media and say anything there and negative news travel faster than positive news.

<Internals\\KIIs\\C-FDA members\\L-KII-35yr old female-FDA-01> - § 1 reference coded [3.69% Coverage]

Reference 1 - 3.69% Coverage

So Probably, in our schools, we should introduce a course that allows people to understand clinical trials because drugs can only be developed when they are tested on human beings and there are phases that new drugs go through the and the drugs that we are using today have all gone through experiment, trials, control experiment using human beings and if something like that is happening, it does not mean that researchers are taking undue advantage over you or study participants. So we should start a course like that in our school to start teaching people at a tender age for them to understand otherwise we would grow up as old as our MPs and our so called Scientists and yet they still do not get the concept of clinical trials.
