# Supplementary material for: Strategies to build trust in the conduct of clinical trials: Stakeholders’ views in a qualitative study in Ghana
Source: PLOS Glob Public Health. 2025 Apr 8;5(4):e0003201. doi: 10.1371/journal.pgph.0003201 (PMC11978029; doi:10.1371/journal.pgph.0003201)
Supplement: Attachment — Submitted filename: Response_to_reviewers_comments_auresp_2.docx [file pgph.0003201.s006.docx]

**PGPH-D-24-00458**

**Title:** Strategies to build trust in the conduct of clinical trials. Stakeholders’ views in a qualitative study in Ghana

**Response to reviewers’ comments**

1. Please review your reference list to ensure that it is complete and correct. If you have cited papers that have been retracted, please include the rationale for doing so in the manuscript text, or remove these references and replace them with relevant current references. Any changes to the reference list should be mentioned in the rebuttal letter that accompanies your revised manuscript. If you need to cite a retracted article, indicate the article’s retracted status in the References list and also include a citation and full reference for the retraction notice.

**Response**: The authors have revised the reference list to reflect the suggestion.

Reviewer #1:

1. The manuscript is technically sound and it provides new knowledge in the area of publication

2. Consider recasting the title to something like…. strategies to build trust.....

Response: We thank the reviewer for this suggestion. The title has been revised to now read as follows: **Strategies to build trust in the conduct of clinical trials. stakeholders’ views in a qualitative study in Ghana** (*Please, refer to the title page for this revision*)

3. There seems to be an over emphasis on infectious diseases as the main basis for clinical trials. It would be better to broaden the usefulness of clinical trials to other areas such as non-communicable conditions.

**Response:** We thank the reviewer for this comment. The manuscript has been revised to replace infectious diseases to diseases to take care of all diseases including non-communicable disease as suggested by the reviewer (*These changes rans throughout the revised manuscript*)

4. Line 176, Similar strategy was used in Hohoe Municipality ....is inaccurate because the selection strategy in Navrongo was different from that used in Hohoe#

**Response**: The sentence has been revised to now read as follows: **In the Hohoe Municipality, purposive sampling technique was also used to select community members and opinion leaders** for the interviewers (*Kindly refer to page 9, third paragraph, line 1 for the revision*)

5. May need to declare possible bias in respondents as only those available and willing were recruited. Those unavailable and unwilling to participate could potentially skew the findings. This should be declared as a study limitation

**Response**: We thank the reviewer for this comment. However, in qualitative, people are selected based on their experience regarding the issue under investigation to share their views. Therefore, participants who were recruited into the study equally met the study recruitment criteria and they did share their views appropriately. Thus, we do not think that those who were not available or people who were unwilling to participate in anyway skewed the findings of the study. However, we have added a paragraph on study limitation to read as: **The study has the following limitations. One limitation of this study is that some of the interviews with community members were conducted in the local language (Ewe), tape recorded, transcribed and translated into English. It is possible that the meaning of some statements made in the local language may have been lost in the English translation. However, the interviews were transcribed by people who were native speakers with experience in transcribing qualitative interviews. Any loss of meaning during the translation was thus minimized, thus did not affect the findings of the study. Another limitation is that since the study used purposive sampling, a non-probability sampling method and also a small sample size, the views expressed by study participants are their personal opinions and may not necessarily represent the views of the larger population** (*Kindly refer to page 28, last paragraph for this addition*)

6. It is not clear what verbal translation of the interview guides means. Were the tools systematically translated before data collection? If not, this may need to be declared as a study limitation as the questions could change with different interviewers translating during data collection.

Response: This has been added as study limitation. (*Please refer to the above response to earlier comment or study limitation on page 27 for this information*)

7. Check editorial typos in lines 12, 163. 176, 180, 351, 364

**Response**: We thank the reviewers for identifying these typos made on the manuscript. We have accordingly corrected them.

Reviewer #2:

1. The opening remarks should start with describing what is trust; and a bit of describing what is clinical trials. This is enabling the reader to link the two key issues.

**Response**: We thank the reviewer for the comments. However, definitions of the two concepts have been captured in the manuscript appropriately. The definition of clinical trial has been captured in the introduction to read as: **Clinical trials are part of the most visible components of biomedical research initiatives aim at generating robust empirical evidence about treatments or preventive interventions to improve health outcomes** (*Please, refer to the first paragraph under the introduction, lines 10 to 12 for this information*)

Trust has also been defined in the manuscript to read as follows: **Trust is viewed as self-assured positive expectations concerning another person’s conduct whereas distrust is having doubts about another person’s conduct** (*Please, refer to second paragraph, page 4, lines 2 and 4 for this information*)

The authors of this study did not want to start with the definitions, but to build a point before the definition. That is why the definitions of the two concepts are not found at the beginning of the introduction. We hope the reviewer will agree with our presentation style.

2. In addition, we have noted trust can also be achieved by clinical trials Sponsor organizations clinical monitoring teams during onsite monitoring of clinical trials and site teams trainings/support. They build trust with regulators and site staff in terms of trusting the data collected and processes.

**Response**: We agree with the reviewer on this point as well. However, these monitory visits or regulatory bodies are there to regulate the conduct of research especially clinical studies and yet there are still issues of trust regarding their conduct. For instance, the negative meaning and rumours associated with the conduct of clinical trials regarding their “experimental” nature often affect trust and the involvement of community members in biomedical research. Also, lack of trust in the design and implementation of clinical trials undermine their successful conduct as was the case in the failed EVD vaccine trial in Ghana. This has informed the design of the study to recommend trust building strategies to improve the conduct of clinical trials especially in developing countries such as Ghana.

3. Misconceptions on drawing blood in northern Ghana as referenced on 17,18 – please add example for the readers to link those misconceptions and the research results.

**Response**: A sentence has been added to give a clear picture of the perceptions people have about blood samples to read as: **Some people perceived that blood samples are taken from clinical trial participants and sold or being used for rituals purposes** (*Please, refer to page 4, second paragraph, the last two lines for this addition*)

4. Study design and site selection - please provide more details why participants were drawn from those particular areas and not randomly across the country, to provide a good representative sample, and avoid bias. May be justification around budget constraints and logistics may suffice. Add a map of Ghana and indicate the study area to the paper. This will make the reader to a clear view of sampling approach. An attempt to conduct phone interview with those leaders and researchers away in rural parts of Ghana may have provided a rich information around remote areas perceptions.

**Response**: We thank the reviewer for the comment. The justification for selecting the study sites is as follows:

**Justification of study sites**

**The reason for selecting KNEM and KNWD was that the two districts fall under the research activities and catchment area of the Navrongo Health Research Centre (NHRC). The NHRC was established in 1989 with the main aim of conducting high quality demographic and health research to inform health policy and practices. Over the years, the centre has conducted many community-based researches, including clinical trial studies in the area with most of them being phase III and phase IV clinical trials. Thus, Community members (i.e those who participated in clinical trials) and opinion leaders were selected and interviewed. Also, some of the key informant interviews with clinical trial scientists and regulators were conducted in the KNEM and KNWD.**

**The failed ebola vaccine trial in Ghana was planned to be conducted in the Hohoe Municipality. The Municipality was therefore selected for this study to explore views of community members and opinion leaders who were either consulted and/ or engaged about the faild ebola vaccine trial. Greater Accra Region is the administrative capital of the republic of Ghana where most stakeholders, political leaders including the members of parliament (MPs) live and work. Therefore, the Region was selected as one of the sites where some of the MPs were selected and interviewed. Also, some of the key informant interviews with clinical trial scientists, regulators and monitors were conducted in Accra. Kintampo Health Research Centre (KHRC) is one of the three health research centres of the Research and Development Division of the Ghana Health Service. The centre was established in 1994 with the main aim of conducting high quality public health and clinical research to influence local and international health policy and programme. Thus, some of the interviews with clinical trial scientists were conducted at KHRC** (*Kindly refer to the methods section, pages 6 and 7 for this addition)*

5. Study population – please provide more details on the composition of the opinion leaders reached out. whether they are church leaders, community advisory board members, local administration etc. This will provide readers with an opportunity to determine the study population of interest if techy want to replicate the study,

**Response**: **Opinion leaders such as chiefs, elders and group leaders were interviewed**. This information has been added to the revised manuscript (*Please, refer to page 7, last paragraph, line 4 for this addition*)

6. Bullet 204 – letters MPs has been used but not defined anywhere else within the paper. Please provide full names for the acronym.

**Response**: This has been corrected and a full definition was given the first time it was used (*Kindly refer to page 7, first paragraph, line 5*)

Generally the study and paper well done. The researchers may in future want to know the if the clinical trials centres staff have trust with studies they are been hired by Investigators to conduct; and also the Investigators views on the studies they are conducting on behalf of the Sponsor companies. This is because, you can inspire confidence and trust on others when yourself not confident in a process. Most of these site staff and Investigators may be motivated by money; and may not trust what they involved in.

**Response**: We thank the reviewers for the suggestions. We hope that the responses provided to the comments are satisfactory and looking forward for formal acceptance and publication of the manuscript to improve trust in the conduct of clinical trials.
