# Supplementary material for: Strategies to build trust in the conduct of clinical trials: Stakeholders’ views in a qualitative study in Ghana
Source: PLOS Glob Public Health. 2025 Apr 8;5(4):e0003201. doi: 10.1371/journal.pgph.0003201 (PMC11978029; doi:10.1371/journal.pgph.0003201)
Supplement: Attachment — Submitted filename: Response to reviewers comments.docx [file pgph.0003201.s005.docx]

**PGPH-D-24-00458**

**Title:** Trust building strategies to improve the conduct of clinical trials. stakeholders’ views in a qualitative study in Ghana

**Response to reviewers’ comments**

Reviewer #1: well written manuscript
logical and need for trust of clinical trial process in present situations
Implementation of these policies dire required for gaining trust of people involved
Most of the clinical trial scenario is wisely presented in the article
I would appreciate the presentation of facts in the article

**Response**: We thank the reviewer for the positive comment. However, the reviewer said he would appreciate the presentation of facts in the article. The manuscript has been revised and we are sure that the reviewer’s concern has been addressed.

Reviewer #2: The Abstract is clearly presented and accurately outlines the study to be presented.

**Response**: We thank the reviewer for the positive comment.

Introduction: is brief but sets the scene, relating to clinical trials in Ghana, for the study to follow. Having read the entire paper I would suggest that this needs to include more background information about the infra-structure to support the conduct of trials in Ghana as what is being proposed about the challenges of conducting research in Ghana are actually globally applicable, it’s the solutions that appear to need to be introduced/ refined.

Response: We thank the reviewer for the suggestion to include in the introduction, infrastructure to support clinical trials conduct in Ghana: A statement has been added in the introduction to read as follows: “**The development of infrastructure in Ghana to support the conduct of clinical trials included the establishment of clinical trial regulatory bodies at the national level such as the Ghana Health Service ethics Review committee and Ghana Food and Drug Authority (FDA). These bodies are mandated to regulate the conduct of clinical trials in Ghana, review and approve trial protocols prior to implementation. They are supposed to conduct a close monitoring and scrutiny of trials to ensure safety of trial participants. Also, there are three health research centres located in the northern, middle and southern belts mandated to conduct high quality health research including clinical trial to inform health policy and practices. Over the years, these trials have helped to improve clinical trial infrastructure and capacity of staff at the three health research centres. Infrastructure such as laboratories to perform various clinical tests, clinical trial unit equipped with consulting rooms, specimen collection rooms, office and twenty-four-hour internet access availability”.** *(Kindly refer to page three, last paragraph for this addition)*

Methods: these are clearly and thoroughly explained with the exception of the Interview Schedule. We are only told that ‘interview guides were developed and used to conduct the interviews…’ How were they developed, who developed them, was there stakeholder involvement in this process?

**Response**: The paragraph has been revised to include a statement on how the interview guide were developed and used to conduct the interviews as follows: **“In-depth interview and key informant interview guides were developed by the first and second authors and reviewed by the other co-authors. The guides were finalised by the first author and used to conduct the interviews with participants”**. (*Kindly refer to page 9 of the revised manuscript for the addition*)

The schedule needs to be included, possibly as supplementary material, (unless I missed it) as it is not possible to gauge whether the interview questions were neutral or biased. The authors clearly wish to make the point about trust being important in decision making but there are other factors that might also impact on decision making yet we do not know if they were included. After all the diligence given to the conduct of the study this is an important omission.

**Response**: We appreciate the concern raised by the reviewer. However, trust was used as the key depended variable in this paper. Other factors affecting decision of people to take part in clinical trials were captured in the first paper published, which has been referenced (<https://doi.org/10.1371/journal.pgph.0001178>) (*Kindly refer to the study design, second paragraph, last line*).

Data analysis: appears to be thorough and guided with reference to established methods.

**Response**: We thank the reviewer for the positive comment.

Results:
Many of the points raised in the results are not specific to Ghana. It is well known that recruitment is a difficult aspect of running a clinical trial and that many studies are eventually seriously under-powered or indeed stopped due to failure to recruit. While the context is different the issues are the same. Why would any potential participant take part in a study if they did not trust the researchers?

**Response**: We thank the reviewer for the comments. Yes, recruitment is a difficult aspect of running clinical trials and that brings to the point about issues of trust and distrust. If the person does not trust the researchers and what they want to do, the person will not take the decision to take part and all that is about the issue of trust. The main aim of this papers has been to explore views of stakeholders about trust and trust building strategies to improve participation in the conduct of clinical trial studies in Ghana. Therefore, the views captured in the paper point to various strategies that participants believed could be employ to address the issue of trust in clinical trial conduct in Ghana.

Page 13 reports the need for intensive stakeholder engagement and this applies to all studies that involve medicinal products. There is no mention of PPI – patient and public involvement, the importance of which has been emphasised in research for many years. (Patient and public involvement policy | Public Involvement Programme | Public involvement - putting you at the heart of our work | NICE and the public | NICE Communities | About | NICE). The programme of research relating to interventions such as the Qunitet Recruitment Intervention (QRI) have been developed in recognition that many studies require specific inputs and processes in order to recruit to target.
(Optimising recruitment and informed consent in randomised controlled trials: the development and implementation of the Quintet Recruitment Intervention (QRI) - PubMed (nih.gov)). These are just two examples of the wider recognition that stakeholder involvement is widely known to be vital in clinical research.

**Response**: The stakeholder engagement mentioned by participants as one of the trust building strategies in clinical trial conduct include community members, patients/potential trial participants, policy makers and civil society organizations in Ghana. If all these people are engaged especially community members to understand the procedures and rationale for clinical trial conduct, it could be a starting point to improve trust in clinical trial conduct in Ghana. The discussion section has been revised to clarify this. (*kindly refer to page 23, second paragraph, lines 1 to 4 for this addition*)

I think that the more important issues are that in Ghana, what are the solutions and what are the barriers and the facilitators?

**Response**: The reviewer also made a comment about the barriers and the facilitators earlier. As pointed out, this paper or work was part of a broader study conducted to look at the issue of trust in clinical trial conduct, factors affecting trust and trust building strategies to improve clinical trials conduct in Ghana, which takes care of the barriers and facilitators mentioned by the reviewer. Please, this is the link to the paper <https://doi.org/10.1371/journal.pgph.0001178>)

P14- recommends the need for public education for clinical trials- I think the important issue here is whose responsibility is it and who will pay for it rather than that it is required.
Similarly ‘…people with communication skills should be engaged to educate the general public about the need for trials to be conducted’ the more interesting question is who should be doing this in Ghana and how and who will fund it?

**Response:** Regarding the funding source for the public education, this is a valid point raised by the reviewer. However, the participants did not mention the funding sources for these activities and that could be a limitation. However, the discussion section has been revised to take care of this. (*Kindly refer to page 24 of the revised manuscript*)

P15- lines 366- 370 - this quote indicates the need for Steering Groups involving opinion leaders, this would be expected in a trial, so the pertinent issue is whether they (SGs) are part of the study protocol in Ghana and if not then they need to include them.

**Response:** These groups are not part of the study protocols in Ghana. That is the reason why participants called for engagement with them to help them have better understanding about clinical trials protocols or designs.

P16 deals with ‘Thorough protocol review procedures’ this puzzles me as surely in order to get funded these clinical trials are reviewed thoroughly. How can the researchers get their study funded without rigorous review?

**Response:** This point made by participants refers to the ethics review processes before the implementation of clinical trial studies. The need for ethics or regulatory bodies to spend much time reviewing clinical trial protocols and making sure that the design and study procedures are in line with ethics principles guiding the conduct of clinical trials. Where there is need for additional information or clarification on certain things in the protocol, the ethics members need to point out all these issues for the investigators to explain before approval. This is different from the review processes between the funder and clinical trial investigators before the funding is awarded. The issue is that, some investigators would usually relax certain issues stated in the protocol because they want to get the funding to conduct the study. It is therefore the duty of regulatory bodies to make sure that things are done based on ethics principles.

The need for ethical approval is raised and again this is a stage that is mandatory for clinical research – I am wondering how have these researchers been able to proceed without ethical approval? There other issues in this section relate to research governance and regulatory processes and these are widely regarded as standard requirements, if they are not part of the research scene in Ghana then I think that a more interesting paper is what is to be done to get them established.

**Response:** There are regulatory bodies in Ghana that are supposed to regulate, review and approve research protocols including clinical trials protocols. Kindly refer to the introduction section of the revised paper for this addition based on the reviewer’s earlier comments for such information to be added to the introduction.

P17 – Line 416 – ‘I will recommend that clinical trial regulators should undergo continuous training …’ once again I don’t think anyone would disagree with this statement, the issue is why is it not happening in Ghana? What are the barriers and facilitators to such training? Good clinical practice certificates are required of researchers in the Uk to be renewed every three years and I believe there are similar requirements in other countries, it would seem that these are also needed in Ghana. If they are not required, what needs to happen for them to be part of the research culture.

**Response:** This is another valid point raised by the reviewer, which in our view need another study to point out issues related to that and what could be done to address the situation. The reason why participants recommended that training be organized for clinical trial regulatory bodies especially anytime news members are recruited is that the training will help them to have better understanding about ethics principles regarding the conduct of clinical trials. This will enable them to adequately review such protocols.

P 18 – lines 432 – 434 – ‘ …it was not about making a lot of promises to convince people to participate ….’ This would be expected and clearly explained in patient information documents / tools etc.

**Response:** Yes, this information is usually included in the information sheet for participants. So, investigators can tell participants that they will do A, B and C for participants when they take part in the study. The investigators may not go by their words when they finally recruit them to take part in the trial. This was reported as one of the factors affecting trust in clinical trial conduct in the first paper that was published. This is the reason why participants made this suggestion that it is good for investigators to tell participants what they can do and when they actually fulfil such promises, it could improve community trust in biomedical research.

Line 449 – ‘They advised research scientists to be transparent, sincere and also stick to research protocols as well as ethics guidelines’ This is the expectation, the more relevant question is if it is not happening then why not and how to change the culture.

**Response:** We thank the reviewer for this point. This is similar to the point made above regarding unfulfilled promises made by clinical trial investigators, which affect trust. However, reason why this is not happening, questions were not asked in this study to solicit information regarding these issues. Therefore, these issues need further investigations. We will recommend that if the reviewer can get the first paper (<https://doi.org/10.1371/journal.pgph.0001178>) on factors affecting trust in clinical trial conduct, it will help your understanding on the strategies recommended by participants.

P 19 Effective monitoring strategies are called for, as above researchers understand the need for these, that is why there are independent trial monitoring committees, why there is the need for fidelity to the protocol and trial process monitoring.
Similarly in other aspects of the results- the question is not whether these are important, it is why are they not in place in Ghana, what are the barriers and how can they be overcome?

**Response:** That is the reason why participants made the recommendation that if there is effective monitoring by regulatory bodies, it would help to strengthen the process and also making sure that investigators implement clinical trials activities based on what has been started in the protocol.

Discussion – to make this paper have more novel it needs to deal more thoroughly with how can all these valuable points raised in the interviews be implemented in ‘…resource poor settings, especially in sub-Sahara Africa’. The issues that are raised from the interviews such as the need for trust in the research/ers, the need for ethical approval, for trial monitoring, for dissemination to communities etc apply more widely than just in Ghana. For example, the MRC Guidance (A new framework for developing and evaluating complex interventions: update of Medical Research Council guidance - PubMed (nih.gov)) includes ways of planning to deal with many of the important tissues raised in this study – this indicates that these issues are widely applicable. However, the means to overcome them in a country with little resource is particularly challenging. It could be argued that the research should not be funded until the research infra-structure is in place to promote all these important issues raised by the authors.

**Response:** We agree with the reviewer and the discussion section has been revised to take care of this point. (*Kindly refer to the discussion section for these revisions*)

On behalf of all co-authors, we think the comments have been adequately addressed to your satisfaction and we look forward to as positive response and publication of this paper to benefit the scientific community on trust building strategies to improved the conduct of clinical trials in Ghana.
